# Understanding patients reinitiating antiretroviral therapy in two South African districts

**DOI:** 10.4102/sajhivmed.v23i1.1380

**Published:** 2022-04-14

**Authors:** Kate Rees, Melanie Bisnauth, Cara O’Connor, Tshifhiwa Ramvhulela, Nomzamo Vali

**Affiliations:** 1 Anova Health Institute, Johannesburg, South Africa; 2 South Africa and Department of Community Health, Faculty of Health Sciences, University of the Witwatersrand, Johannesburg, South Africa

Antiretroviral treatment (ART) coverage is South Africa’s biggest obstacle to 90-90-90 achievement. Many people living with HIV who are not on ART have started but experienced interrupted treatment. A major programmatic challenge is how to return them to care.

We aim to describe patients reinitiating ART in two districts to inform these efforts.

As part of evaluating a campaign to return patients to care (Welcome Back Campaign), we asked lay counsellors to collect data on all patients reinitiating ART. Data collection started in August 2021 and we report data through September 2021 for two districts, one metropolitan and one district municipality.

Two hundred and seventeen forms were completed, 120 from Cape Town and 97 from Sedibeng. Fifty-eight percent (*n* = 126) of reinitiating patients were women. Thirty-two percent (*n* = 68) had interrupted treatment for three months or less, while 40% (*n* = 85) had interrupted treatment for more than 12 months. The commonest reported reason for interruption in Cape Town was relocation or mobility (27%; *n* = 32), followed by difficulty getting time off work (15%; *n* = 17) and disclosure issues (9%; *n* = 11). In Sedibeng the top reasons were difficulty getting time off work (21%; *n* = 20), relocation (18%; *n* = 17) and long waiting times (12%; *n* = 12). Women were more likely to report disclosure issues and being scared to come back to the clinic as a reason for interrupting, while men were more likely to cite staff attitude. Reasons for returning to care included worry about being off ART (38%; *n* = 82), feeling sick (15%; *n* = 34) and tracing (12%; *n* = 27). Men were more likely to report illness and improved accessibility as a reason for seeking care, while women were more likely to report media messaging.

It is critical that health services are supportive of patients reinitiating ART after interruptions, and that more enabling systems for patients moving between clinics are developed. Nudges should be developed to encourage people already worried about having interrupted treatment to reinitiate it.

The authors would like to thank the Sedibeng and Cape Town Departments of Health.

**Ethical considerations:** This study was approved by the Human Sciences Research Council Human Research Ethics Committee, reference: REC 3/22/08/18.

**Funding information:** This research is made possible by the generous support of the American people through the United States Agency for International Development (USAID) and the President’s Emergency Plan for AIDS Relief (PEPFAR) under Cooperative Agreement number 72067418CA00023 to the Anova Health Institute.

